# Computational modeling of inhibition of voltage-gated Ca channels: identification of different effects on uterine and cardiac action potentials

**DOI:** 10.3389/fphys.2014.00399

**Published:** 2014-10-16

**Authors:** Wing-Chiu Tong, Iffath Ghouri, Michael J. Taggart

**Affiliations:** Institute of Cellular Medicine, Newcastle UniversityNewcastle upon Tyne, UK

**Keywords:** computational modeling, uterus, cardiac, calcium channel, tocolytics

## Abstract

The uterus and heart share the important physiological feature whereby contractile activation of the muscle tissue is regulated by the generation of periodic, spontaneous electrical action potentials (APs). Preterm birth arising from premature uterine contractions is a major complication of pregnancy and there remains a need to pursue avenues of research that facilitate the use of drugs, tocolytics, to limit these inappropriate contractions without deleterious actions on cardiac electrical excitation. A novel approach is to make use of mathematical models of uterine and cardiac APs, which incorporate many ionic currents contributing to the AP forms, and test the cell-specific responses to interventions. We have used three such models—of uterine smooth muscle cells (USMC), cardiac sinoatrial node cells (SAN), and ventricular cells—to investigate the relative effects of reducing two important voltage-gated Ca currents—the L-type (I_CaL_) and T-type (I_CaT_) Ca currents. Reduction of I_CaL_ (10%) alone, or I_CaT_ (40%) alone, blunted USMC APs with little effect on ventricular APs and only mild effects on SAN activity. Larger reductions in either current further attenuated the USMC APs but with also greater effects on SAN APs. Encouragingly, a combination of I_CaL_ and I_CaT_ reduction did blunt USMC APs as intended with little detriment to APs of either cardiac cell type. Subsequent overlapping maps of I_CaL_ and I_CaT_ inhibition profiles from each model revealed a range of combined reductions of I_CaL_ and I_CaT_ over which an appreciable diminution of USMC APs could be achieved with no deleterious action on cardiac SAN or ventricular APs. This novel approach illustrates the potential for computational biology to inform us of possible uterine and cardiac cell-specific mechanisms. Incorporating such computational approaches in future studies directed at designing new, or repurposing existing, tocolytics will be beneficial for establishing a desired uterine specificity of action.

## Introduction

Computational modeling of an action potential (AP) of an electrically excitable cell was first developed in 1952 with the landmark study of neurons (Hodgkin and Huxley, 1952). Its success led to the development of other models such as the tonic AP in cardiac cells (Noble, 1960) and the bursting AP in β-pancreatic cells (Chay and Keizer, 1983). In the intervening years there has been an enormous advance in our understanding of the cardiac physiome (Noble, 2007; Schmitz et al., 2011; Noble et al., 2012) and computational analysis of electrical excitability now runs hand-in-hand with physiological experimentation in heart research (Crampin et al., 2004; Bassingthwaighte et al., 2009; Masumiya et al., 2009; Nikolaidou et al., 2012; Zhang et al., 2012). Many computational models exist to describe in considerable detail cardiac cell-specific excitation-contraction properties, including the biophysical details of the constituent ion currents and calcium fluxes. These include multicellular tissue and organ maps of spatiotemporal electrical and calcium wave propagation (Rudy, 2000; Zhang et al., 2000; Kleber and Rudy, 2004; Severi et al., 2009; Aslanidi et al., 2011b; Atkinson et al., 2011). Mathematical models are continuously being developed and applied to predicting the risks of pathophysiological phenomena (e.g., the likelihood of dyssynchronous activation and fibrillation) (Benson et al., 2008; Bishop and Plank, 2012; Cherry et al., 2012; Kharche et al., 2012; Behradfar et al., 2014) as well as the potential beneficial effects of drugs and treatments (Levin et al., 2002; Muzikant and Penland, 2002; Davies et al., 2012; Mirams et al., 2012; di Veroli et al., 2013, 2014).

The uterus is also an electrically excitable tissue whose contractile function is determined by episodic spontaneous APs and calcium fluxes. Although our comprehension of the electrophysiological basis of uterine AP formation lags behind that of cardiac muscle, there is an increasing awareness that computational approaches, such as those which have been applied so extensively to cardiac muscle, may foster advances in this matter (Taggart et al., 2007; Aslanidi et al., 2011a; Tong et al., 2011; Sharp et al., 2013). For example, we have developed a biophysically-detailed uterine smooth muscle cell (USMC) model validated against experimental data and it can describe many different uterine AP forms and the corresponding intracellular calcium changes (Tong et al., 2011).

Utilizing computational models for the examination of uterine APs offers the additional possibility of predicting the likely actions of drugs that target ion channels or exchangers with the intention of attenuating premature uterine contractions. Hitherto, such tocolytics have been used clinically without prior extensive *in silico* assessment of effectiveness. Uterine APs, and the resultant contraction of smooth muscle cells, are markedly dependent upon the activation of a prominent voltage-gated inward (depolarizing) current, the long-lasting L-type calcium current (I_CaL_). Nifedipine, an L-type voltage-gated calcium channel blocker, is currently used as a tocolytic treatment (RCOG, 2011). This treatment is useful for delaying labor in the short term (Conde-Agudelo et al., 2011). However, it is not without adverse side effects, in particular on maternal cardiovascular performance (van Veen et al., 2005; Guclu et al., 2006; Gaspar and Hajagos-Toth, 2013), and it is not presently recommended for longer-term use, nor for women with cardiac disease (RCOG, 2011). This alerts one to another necessary consideration of tocolytic drugs intended to limit uterine APs namely, what possible actions may there be on cardiac electrical excitability?

Another voltage-gated Ca current, in addition to I_CaL_, that, theoretically, may be a suitable target for developing tocolytic drugs is the short-lasting, transient T-type Ca channel current (I_CaT_). T-type calcium currents are believed to play a role in pacemaking in many cell types (Mangoni et al., 2006; Perez-Reyes et al., 2009). I_CaT_ has been observed in uterine tissues (Inoue et al., 1990; Young et al., 1991, 1993; Blanks et al., 2007) and putative blockers of I_CaT_ reduce *in vitro* uterine contractions (Lee et al., 2009). I_CaT_ has a different current-voltage (I-V) profile from I_CaL_ and, unlike the latter, there is considered to be little I_CaT_ present in ventricular cardiomyocytes although it has been suggested to contribute to sinoatrial (SA) node APs (Ono and Iijima, 2010; Mesirca et al., 2014).

Our over-arching objective was to utilize computational models of uterine and cardiac cells to theoretically test the possible cell-specific effects of inhibiting voltage-gated Ca entry (I_CaL_ and I_CaT_) in a manner that may be anticipated to occur with drugs targeting these pathways. Using publicly-available computational models of uterine and cardiac APs we have performed a series of simulation experiments to investigate the following questions:

- Are there comparable effects on uterine and cardiac APs of reducing I_CaL_?
- Are there similar effects on uterine and cardiac APs of reducing I_CaT_?
- Does combined reduction of I_CaL_ and I_CaT_ have differential actions on uterine and cardiac APs?

## Methods

### Uterine smooth muscle cell model

**Figure 1:**
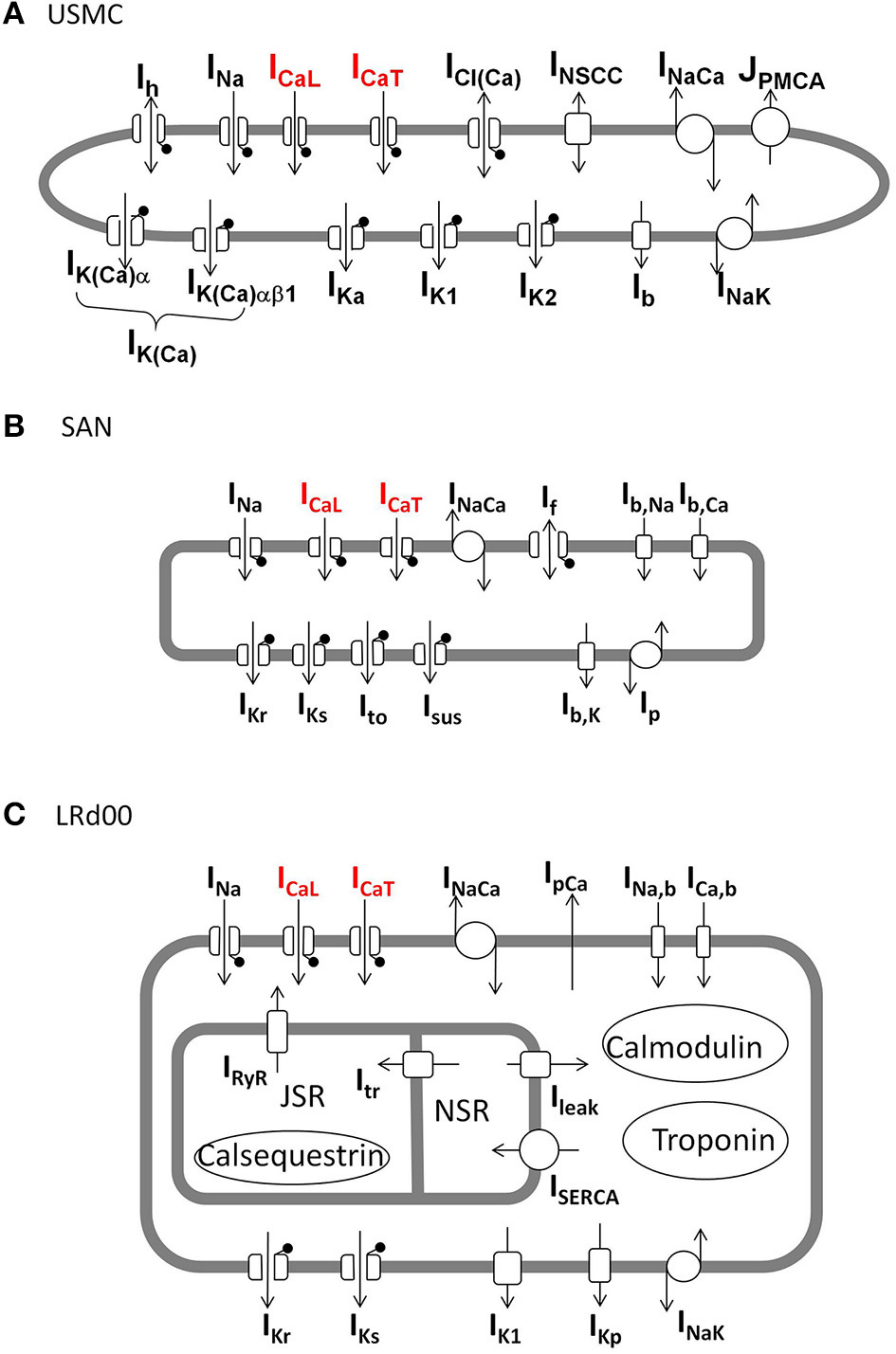
**Schematic diagrams of the ionic currents in each of the three cell models. (A)** Uterine smooth muscle cell model (USMC) (Tong et al., 2011), **(B)** rabbit sinoatrial nodal cell model (SAN) (Garny et al., 2003), **(C)** modified guinea pig ventricular cell model (LRd00) (Faber and Rudy, 2000). The lollipop sign indicates channels that are voltage-gated. The L-type Ca channel and T-type Ca channel, carrying I_CaL_ and I_CaT_, respectively, are indicated in red.

For these simulation studies we used our previously published USMC model (Tong et al., 2011). A schematic of the ionic currents contributing to the model is shown in Figure 1A. The model source code, parameter values and the full description are provided in Tong et al. (2011). All the USMC AP simulations were started at the same resting state. The numerical values of all the dynamical variables at this state—the initial conditions—are provided in the Supplementary Materials.

### Cardiac cell models

There are numerous computational models of cardiac cells and many are listed in the CellML model repository (http://models.cellml.org/). They represent different parts of the heart from the central and peripheral sinoatrial nodal cells (Zhang et al., 2000; Garny et al., 2003), the atrial cardiomyocytes (Aslanidi et al., 2009), the atrial-ventricular node and His-bundle (Inada et al., 2009), Purkinje fiber cells (Stewart et al., 2009; Li and Rudy, 2011) and ventricular cardiomyocytes (Faber and Rudy, 2000). These cell types can be grouped, rather roughly, into those of a contractile or conducting function. In our preliminary simulations using these cardiac models, we selected the ventricular and sinoatrial nodal cell models that showed the greatest propensity to alter AP form following a reduction of Ca current thus forming the most sensitive situation for comparing the same maneuvre to that on the USMC model.

For the sinoatrial nodal cell, we used the Garny et al. (2003) rabbit sinoatrial nodal cell model (SAN) and its 0D-capable version configurations for the SAN central cell. A schematic of the ionic currents contributing to the model is shown in Figure 1B. The model equations and parameter values and are listed in the CellML model repository. The initial conditions for the SAN model are provided in the Supplementary Materials.

For the ventricular cardiomyocyte cell, we used as a platform the Faber and Rudy (2000) guinea pig cell model (LRd00) and its M cell configurations. A schematic of the ionic current components of the model is shown in Figure 1C. The model equations, parameter values and initial conditions are listed in the CellML model repository. A copy of the model source code can also be downloaded from http://rudylab.wustl.edu/research/cell/methodology/cellmodels/LRd/code.htm. However, as detailed below in the Results, we had to modify this model in order to incorporate a more robust description of I_CaT_. The LRd00 modified model was paced for 20 min at 2 Hz to allow it to reach a stable state with its M cell configurations. Then, the values of all the dynamical variables in this stable state were saved: these stable state conditions of the modified LRd00 model are provided in the Supplementary Materials.

### Simulations of uterine and cardiac APs with reduced I_CaL_ and I_CaT_

The effects of reduced I_CaL_ and I_CaT_ on the uterine and cardiac APs were assessed and quantified. The I_CaL_ and I_CaT_ were reduced by multiplying their maximal conductances with a scaling factor between 0 and 1 and we assumed the same proportional reduction would occur in all three cell types. As the APs between the three unique cell types have different forms and characteristics, it is difficult to use a common measure to assess their AP behaviors. Therefore, we chose to assess a characteristic that best reflects the main function of each of the cell types: the bursting activities in the uterine cell, the pacemaking ability of the SAN cell and the action potential duration (APD) of the ventricular cell. For the USMC model, after adjusting the levels of I_CaL_ and I_CaT_, an AP was induced by a 5 s current clamp at −0.5 pA pF^−1^ and the maximal peak membrane voltage (V_peak_>) reached after the first initial spike was measured. This assessment gives an indication of the presence or absence of bursting spikes in a USMC AP. For the SAN model, after adjusting the levels of I_CaL_ and I_CaT_, the model was simulated for 10 s and the pacemaking frequency was measured from the APs in the last 2 s of simulations. For the LRd00 model, an AP was induced by a 0.5 ms current pulse at −90 pA pF^−1^ and the APD at 90% repolarization level (APD_90_) was assessed.

All simulations were computed using XPPAUT Version 6 (Ermentrout, 2002) in a Dell Optiplex PC with an Intel(R) Core(TM) i7-3770 CPU @ 3.40 GHz. For the USMC model, all simulations were computed with a fixed time step of 0.02 ms and the Euler method. For the SAN model, all simulations were computed with a fixed time step of 0.1 ms and the fourth-order Runge–Kutta method. For the LRd00 model, all simulations were computed with a fixed time step of 0.002 ms and the fourth-order Runge–Kutta method.

## Results

### Validation of the cell models

**Figure 2:**
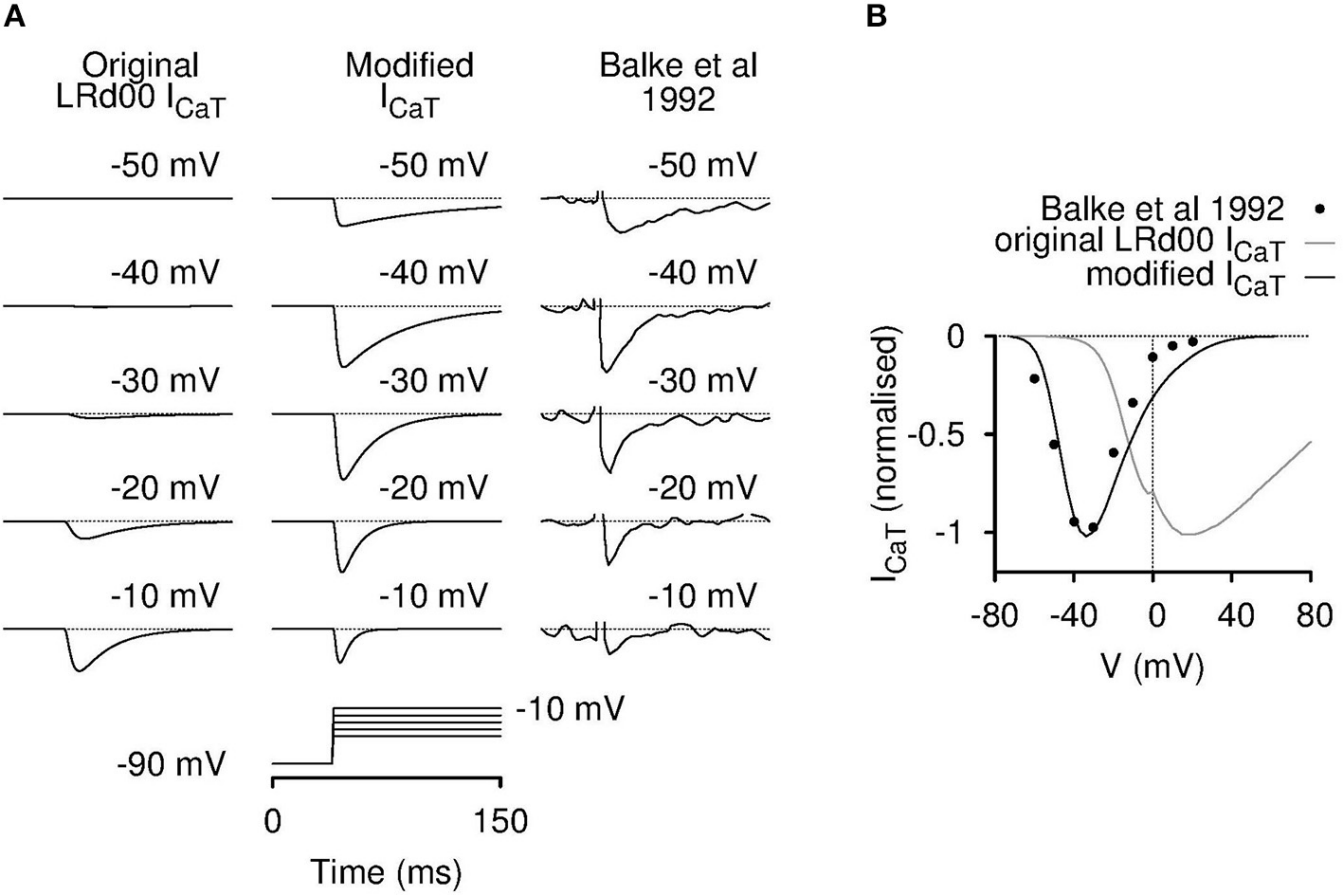
**Incorporation of a modified T-type calcium current model for LRd00**. The original I_CaT_ contained in the LRd00 cardiac ventricular cell model did not behave like a T-type calcium current. The simulated raw current tracings **(A)** and the I-V current plots **(B)** appeared at holding membrane potentials farther to the right than of the experimental data of Balke et al. (1992) that was used as a source experimental dataset. Simulated current tracings at five different voltage steps from a holding potential of −90 mV **(A)** and simulated peak current-voltage (I-V) relationships **(B)** of the original and modified I_CaT_ currents are shown in comparison to the experimental data from Balke et al. (1992). All current tracings were normalized to their maximal peak current from their I-V relationships. The modified I_CaT_ now closely resembled the experimental data. (Experimental tracings and data, adapted with permission from Figure 4 from Balke et al. (1992); copyright 1992 The Physiological Society.)

We first verified each source code of the three individual cell models with their respective published results. Both the USMC and the SAN models were validated in this regard. Surprisingly, we found that the LRd00 model could not be validated so in respect of I_CaT_: the simulated current tracings (Figure 2A), and the corresponding current-voltage (I-V) relationship (Figure 2B), of the LRd00 model did not match the experimental results that they were rated against (Balke et al., 1992). The simulations produced current responses at membrane voltages that were much farther to the right (positive) than observed in the experimental data or, indeed, to that anticipated from other I_CaT_ models. When we traced back the original formulation of this I_CaT_ to Zeng et al. (1995), and the experimental values from which its kinetics details were based to Droogmans and Nilius (1989), we did not find any typographical errors in the model equations or the experimental values to explain the discrepancy. As an aim of our study was to investigate the effects of altering I_CaT_, it was essential that the models incorporated the correct formulations. We were left, therefore, with no option but to attempt to modify the I_CaT_ in the LRd00 model such that it *did* reflect more closely the experimental data. For those interested in the details of this process, our attempts to modify the model were undertaken as follows: we had first tried, but failed, to produce a reasonable I_CaT_ model by either adjusting the LRd00 I_CaT_ equations or reformulating new equations using data from Droogmans and Nilius (1989). We next tried substituting the LRd00 I_CaT_ with a validated I_CaT_ model from Li and Rudy (2011), which was developed for canine Purkinje fiber cells. The simulated current tracings with this canine I_CaT_ resemble the I_CaT_ current tracings in Balke et al. (1992) but the kinetics were too fast and the peak of the I-V relationship was still too positive compared to the experimental data from Balke et al. (1992). Using the I_CaT_ from Li and Rudy (2011) as our template, we corrected the differences between the simulations and the experimental data with the modifications that resulted in Equations (1–8) for the new ventricular I_CaT_ that are listed in the Supplementary Materials. As discussed by Droogmans and Nilius (1989), we also found that the kinetics of the ventricular I_CaT_ currents were best described by a gating product of b^2^g where b and g are gating variables for activation and inactivation (Equation 1 in the Supplementary Materials). The I-V data in Balke et al. (1992) was matched with a half-activation at −50 mV in the activation steady-state function (Equation 3 in the Supplementary Materials). Although this half-activation value deviated from the reported value for I_CaT_ (Droogmans and Nilius, 1989), it is similar to those reported values from clonal Cav3.1 expression data (Serrano et al., 1999; Hering et al., 2003). As the kinetics of the canine I_CaT_ model were too fast compared to the ventricular cell experimental data within the LRd00 model (i.e., the data from Balke et al., 1992), we slowed down the activation and inactivation by scaling the time constants (Equations 5, 6 in the Supplementary Materials). The resultant new I_CaT_ model satisfactorily described the ventricular I_CaT_ data (Balke et al., 1992) and we replaced the original LRd00 I_CaT_ with this new I_CaT_ model. The ratio between peak I_CaT_ and I_CaL_ in guinea pig ventricular cells are reported to be between 0.13 and 0.19 (Balke et al., 1992; Masumiya et al., 1998; Zorn-Pauly et al., 2004). Therefore, we chose a value for the maximal conductance of our new I_CaT_ model (g_CaT_) so that the ratio of the maximum I_CaT_: I_CaL_ in the modified LRd00 cell model was 0.15.

### Normal USMC, SAN, and ventricular APs

USMC APs can take a variety of forms of variable durations. Bursting type USMC AP, wherein an initial spike of an AP followed by rapid and repetitive fluctuations in membrane potential that persist for the whole AP duration, are often observed. These may assist in the maintenance of long-duration contractions of many tens of seconds that are a feature of the uterus during labor. Therefore, a promising feature of a tocolytic would be one that could dampen, or delay, the bursting in the USMC APs with minimum effects on the APs of the cardiac SAN and the ventricular cells.

**Figure 3:**
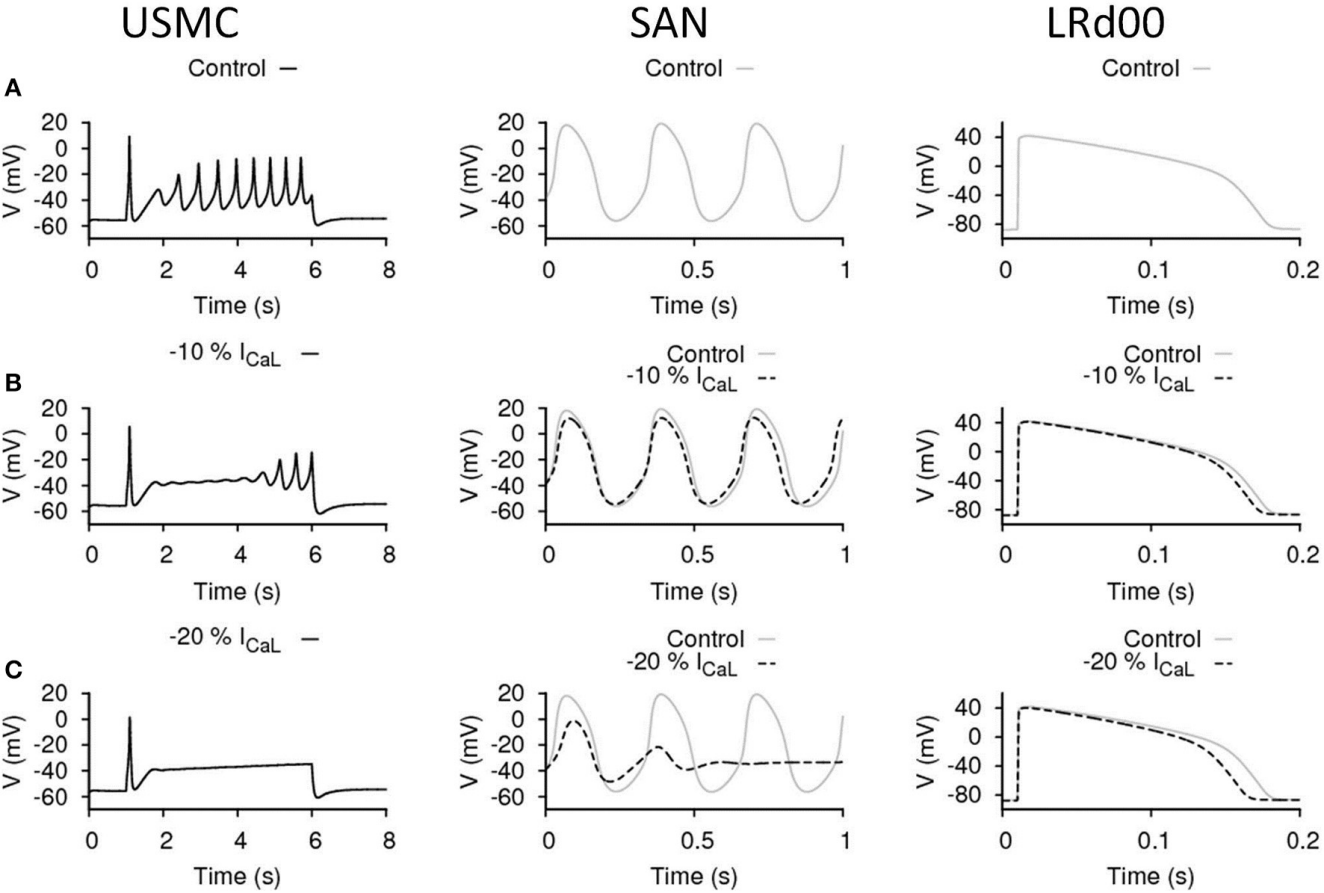
**Effects of reducing I_CaL_ in uterine and cardiac cells**. Effects on the action potential forms and characteristics with reduced I_CaL_ in different cell types: the USMC cell model (left), the SAN cell model (center), and the LRd00 ventricular cell model (right). **(A)** Control configuration at full I_CaL_ and the effects of reducing I_CaL_ by **(B)** 10% and **(C)** 20%. The negative sign indicated reduction.

Figure 3A shows the simulated APs from the three cell models under control conditions before any maneuvre. The USMC model showed a resting membrane potential (RMP) of −55.5 mV before stimulation. During the evoked USMC AP, burstings occurred throughout the AP with a frequency between 2 and 2.54 Hz and the amplitudes of the repetitive spikes were around 40 mV. The V_peak_> after the initial spike was −7 mV. The SAN cell is autorhythmic where periodic APs occur without external stimulation. The frequency and amplitude of the pacemaking activities were 3.15 Hz and 75 mV (between −56 and 19 mV), respectively. These values were the same throughout the whole 10 s simulation. The LRd00 ventricular cell showed a comparatively hyperpolarized (negative) RMP at −87 mV. A single AP was evoked in respond to a stimulus and the ventricular APD_90_ was 165 ms. These quantified characteristics are the reference values before any maneuvre.

### Effects of reducing I_CaL_ in uterine and cardiac cells

I_CaL_ is a major depolarization current in USMCs and thus a logical target for tocolytics. We examined the effect of reducing I_CaL_ by 10% (Figure 3B). In the USMC model, this change dampened the initial repetitive spikes of the USMC AP and reduced the duration of the burstings by around 60%. The amplitudes of the remaining, late-onset, bursting spikes were reduced by 30% to about 28 mV and the V_peak_> of the USMC AP was lowered to −14.8 mV. A less severe effect of 10% less I_CaL_ was evident in the APs from the SAN and the LRd00 models. The amplitude of the SAN pacemaking APs was modestly reduced by 12% and the frequency remained unchanged. The LRd00 APD_90_ was slightly reduced by 5%. When I_CaL_ was reduced by 20% (Figure 3C), the burstings of the USMC AP were suppressed completely. However, the SAN cell also stopped pacemaking. The LRd00 APD_90_ was slightly reduced by ~10% at 150 ms. These data indicated that although reducing some I_CaL_ suppressed uterine bursting APs, this maneuvre also affected both the cardiac pacemaker APs and the ventricular AP.

### Effects of reducing I_CaT_ in uterine and cardiac cells

**Figure 4:**
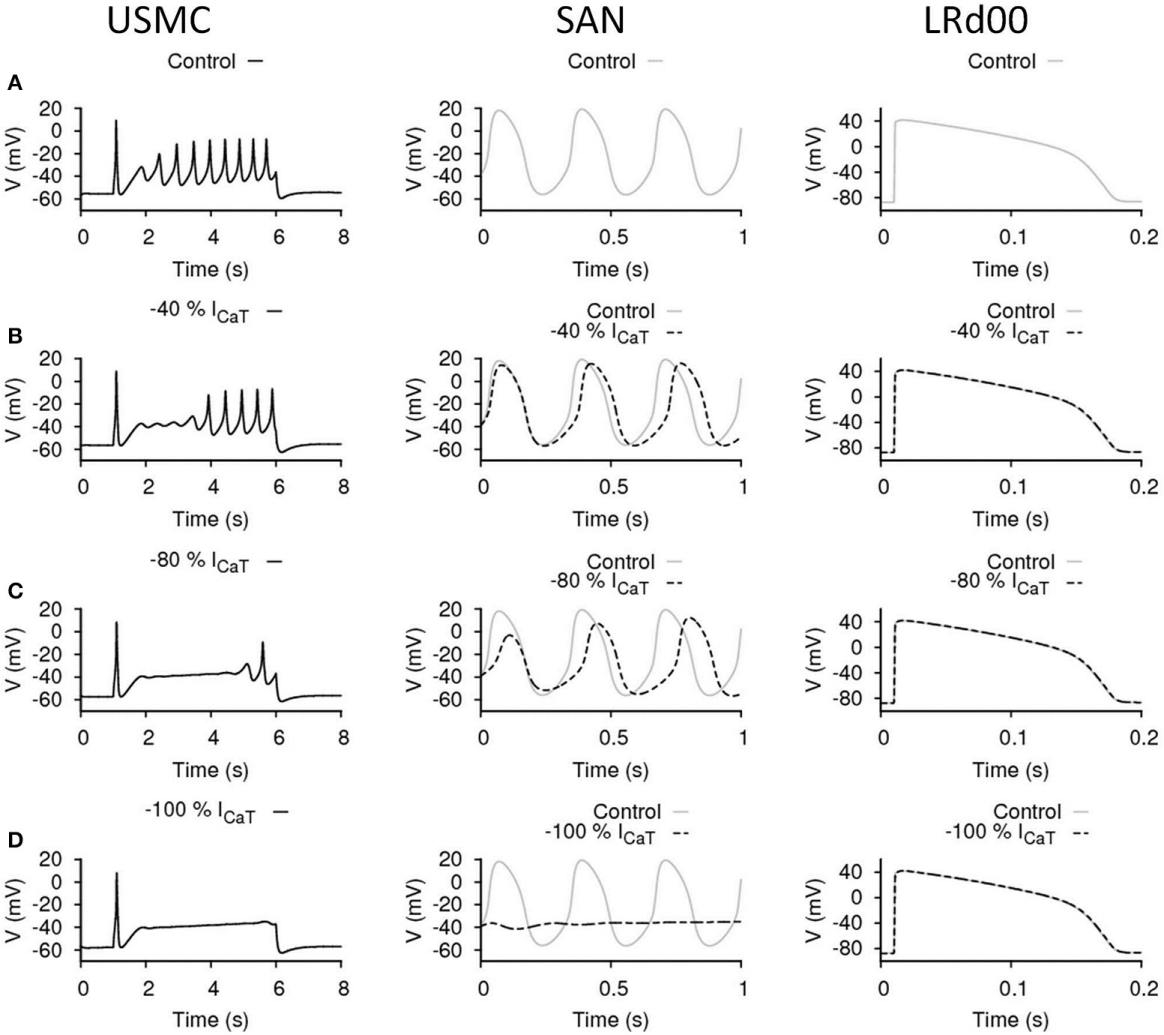
**Effects of reducing I_CaT_ in uterine and cardiac cells**. Effects on the action potential forms and characteristics with reduced I_CaT_ in different cell types: the USMC cell model (left), the SAN cell model (center), and the LRd00 ventricular cell model (right). **(A)** Control configuration at full I_CaT_ and the effects of reducing I_CaT_ by: **(B)** 40%, **(C)** 80%, and **(D)** 100%. The negative sign indicated reduction.

I_CaT_ may be one of the SAN pacemaking currents in the heart and may also be involved in the generation of APs in USMCs. The effects of our maneuvres to reduce I_CaT_ in each of the three cell types are shown in Figure 4. The control cases of the three models are displayed in Figure 4A. Reduction of I_CaT_ by 40% (Figure 4B) delayed the onset of the burstings in the USMC AP. However, both the V_peak_> and the amplitude of the later-onset burstings remained the same as the control. With 40% less I_CaT_, the pacemaking by the SAN cell slowed down to about 2.94 Hz with the peak potential at a slightly lower level of 15 mV. Reduction of I_CaT_ by 40% did not affect the LRd00 APD_90_. When more I_CaT_ was reduced, by 80% (Figure 4C), the onset of the USMC burstings was further delayed with only one spike appearing at the end of the AP. However, the amplitude of this spike was similar in size to those of the control. Also, the RMP level became more hyperpolarized at ~−57 mV. With this large reduction of I_CaT_ (by 80%), the SAN pacemaking APs slowed down to 2.63–2.7 Hz. The pacemaking potential in the SAN model remained at the same level at −56 mV but the peak potential was lowered to 12 mV. At this level, the SAN model also showed a longer (~0.5 s) transient during simulation. With 80% less I_CaT_, the LRd00 APD_90_ remained unchanged. Without I_CaT_ (Figure 4D), the RMP level of the USMC became more hyperpolarized at ~−58 mV and the bursting of the USMC AP stopped. The SAN pacemaking also ceased without I_CaT_ but the LRd00 AP was unaffected. These data indicated that reduction of I_CaT_ suppressed the uterine bursting AP with no impact on the ventricular AP. However, depending on the magnitude of I_CaT_ reduction, there was also an effect on the SAN APs.

### Effects of reducing I_CaL_ and I_CaT_ in uterine and cardiac cells

**Figure 5:**
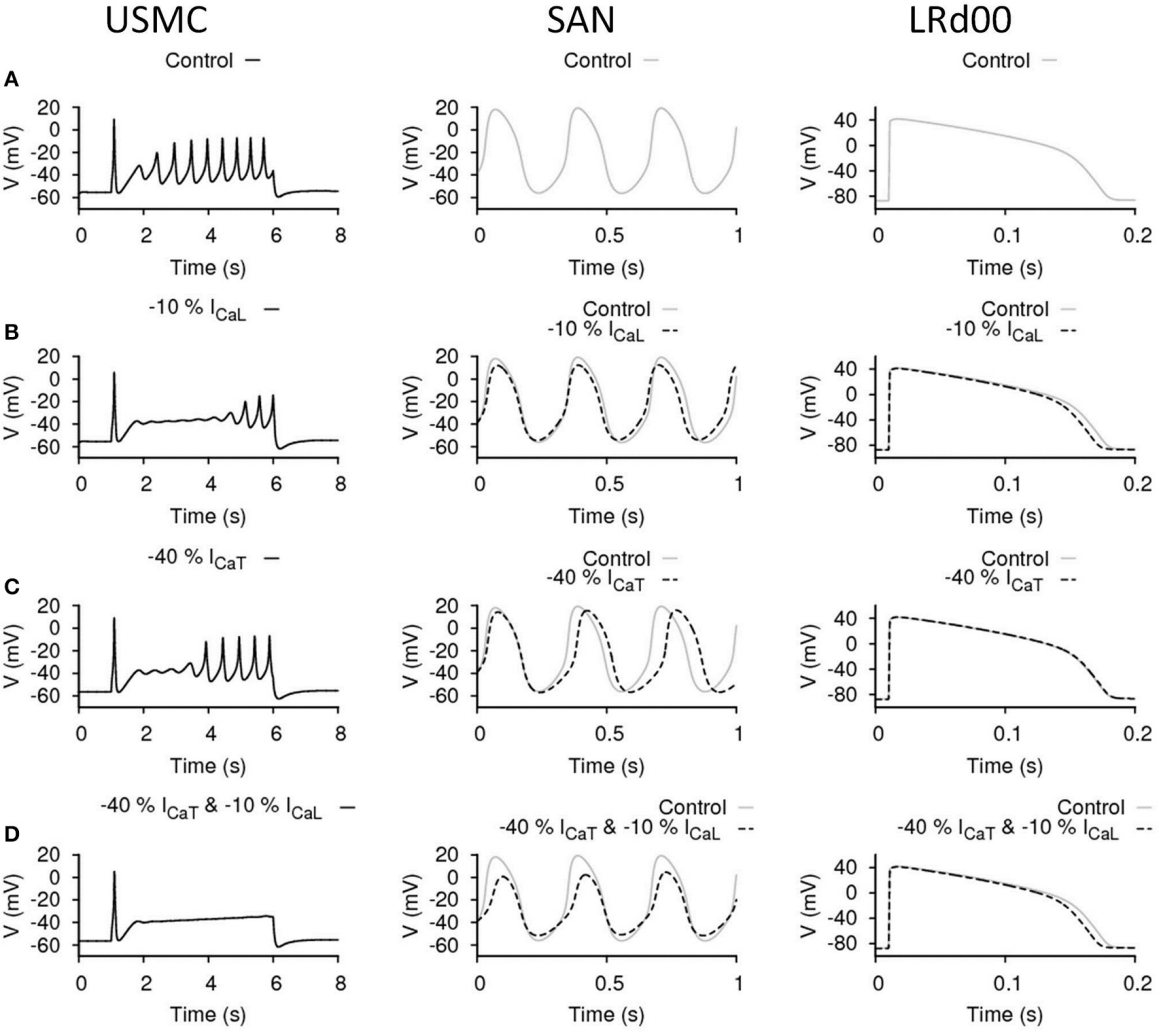
**Effects of reducing I_CaL_ and I_CaT_ in uterine and cardiac cells**. Effects on the action potential forms and characteristics when both I_CaL_ and I_CaT_ were proportionally reduced in different cell types: the USMC cell model (left), the SAN cell model (center), and the LRd00 ventricular cell model (right). **(A)** Control configuration before reducing either of the currents. **(B)** Reducing I_CaL_ by 10% only. **(C)** Reducing I_CaT_ by 40% only. **(D)** Reducing I_CaL_ by 10% and I_CaT_ by 40%. The negative sign indicated reduction.

The above data suggested that there was some promise in reducing either I_CaL_ or I_CaT_ and effecting an inhibition of USMC APs with little deleterious action on ventricular APs. The manouevres that reduced I_CaL_ or I_CaT_ and had mild effects on SAN AP may be tolerable. However, reductions in either current that effected a larger inhibition of uterine APs, as one may desire the action of a tocolytic in our experimental setting to be, also had dramatic actions on SAN AP form. The outcomes of reducing I_CaL_ or I_CaT_ in the USMC models were slightly different: reducing I_CaT_ altered the RMP and the onset of spike bursting whereas changes in I_CaL_ had a predominant action on spike amplitude. This begat the question, could a combined reduction of I_CaL_ and I_CaT_ exert the desired action on USMC APs with little impact on cardiac cell APs? An example is illustrated in Figure 5. The control cases of the three models are shown in Figure 5A and the effects of reducing only I_CaL_ by 10%, or only I_CaT_ by 40%, are illustrated again in Figures 5B,C, respectively. This enables comparison to the effect arising when both I_CaL_ and I_CaT_ were reduced, by 10 and 40%, respectively, (Figure 5D). In this case, the burstings of the USMC AP were completely suppressed (Figure 5D). With this combination, the pacemaking frequency of the SAN APs remained the same at 3.125 Hz and the amplitude was reduced by 20% (from −52 to 7.7 mV). The LRd00 APD_90_ was slightly reduced by ~4% to 158 ms. Compared to the cases when only either I_CaL_ or I_CaT_ were reduced alone to completely suppress the burstings of the USMC AP (Figures 3C, 4D), this combined approach clearly performed better while preserving many of the properties of the cardiac APs.

**Figure 6:**
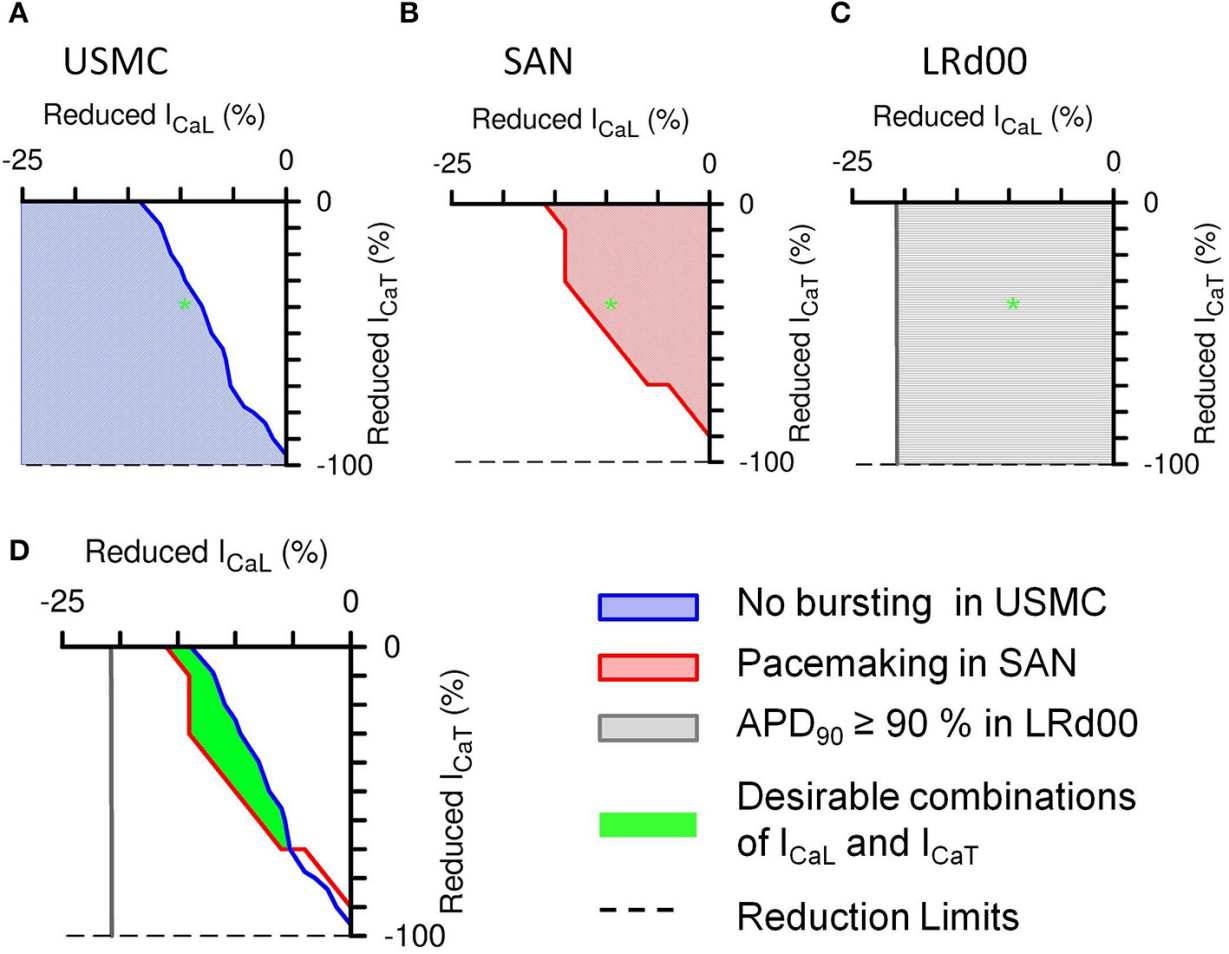
**Summarized effects of different combinations of reduced I_CaL_ and I_CaT_ on the APs of the three different cell types**. The shaded areas in the 2-parameter maps indicate the type of AP that would be produced with the different combinations of proportionally reduced I_CaL_ and I_CaT_: **(A)** suppressed bursting in the USMC cell model, **(B)** pacemaking activities in the SAN cell model, and **(C)** less than 10% change in the action potential duration at 90% repolarization level (APD_90_) in the LRd00 ventricular cell model. The green star illustrates the corresponding position on each 2-parameter map of the example shown in Figure 5D, i.e., with reductions of 10% I_CaL_ and 40% I_CaT_. **(D)** Combinations of I_CaL_ and I_CaT_ (green area) that would suppress bursting in the USMC without affecting the pacemaking in the SAN cell or the APD_90_ in the LRd00 ventricular cell.

We plotted two-parameter maps of percentage current inhibitions—of I_CaL_ vs. I_CaT_—in each cell model to examine the effective ranges of different combinations of I_CaL_ and I_CaT_ reductions on the properties of uterine and cardiac APs (Figure 6). After the initial spike of a USMC AP, the peak of the bursting spikes typically reach values between −20 and 0 mV whereas without bursting, the plateau AP voltage usually stayed below −30 mV. Therefore, we assessed the presence or absence of burstings in a USMC AP from the level of V_peak_> when the proportions of I_CaL_ and I_CaT_ were changed and the results of the parameter plots are color-coded in Figure 6. In Figure 6A, the white region indicated the combinations of I_CaL_ and I_CaT_ that would produce a USMC AP with some burstings and the shaded region indicated the combinations of currents with which the USMC cell would not produce such activity. The USMC AP example shown previously in Figure 5D, with 10% reduction of I_CaL_ and 40% reduction of I_CaT_, lay just within the non-bursting domain (as indicated by the green star). For the SAN cell, the pacemaker frequency was monitored as one changed the proportions of I_CaL_ and I_CaT_ and the results are shown in Figure 6B. The colored domain indicated pacemaking occurrence and the white region suggested the parameter combinations would result in no pacemaking. The frequencies of the pacemaking APs were similar throughout the pacemaking zone and the transition between the two regions was steep (not shown). The SAN example shown in Figure 5D with 10% reduction of I_CaL_ and 40% reduction of I_CaT_ was within the pacemaking region. For the LRd00 ventricular cell, the APD_90_ was monitored as the proportions of I_CaL_ and I_CaT_ were changed and the results are shown in Figure 6C. Changes in the APD_90_ occurred only with changes in I_CaL_, indicating that I_CaT_ did not influence the APD of the ventricular cell model. From overlapping these parameter maps, we derived the boundaries of combinations of I_CaL_ and I_CaT_ for attaining specific sets of AP forms in the uterine and cardiac cells as shown in Figure 6D. The colored lines separate different properties of the uterine and cardiac cells obtained from the simulations in Figures 6A–C. The blue line traces the level of V_peak_> at −30 mV in the parameter space of I_CaL_ and I_CaT_ of the USMC cell model and it separates the bursting and quiescent conditions. The red line separates the pacemaking conditions with I_CaL_ and I_CaT_ in the SAN cell model from the non-pacemaking. We allowed for 10% variations in the APD_90_ of the LRd00 ventricular cells and this threshold at 150 ms was indicated by the gray line. These three criteria were superimposed on the same parameter map of I_CaL_ and I_CaT_ and revealed an overlapping area that satisfied all three conditions. In other words, any combination of reduced I_CaL_ and I_CaT_ within this area can produce our desired outcome for tocolytic treatment, namely, effective suppression of USMC APs with minimal effects on cardiac APs.

**Figure 7:**
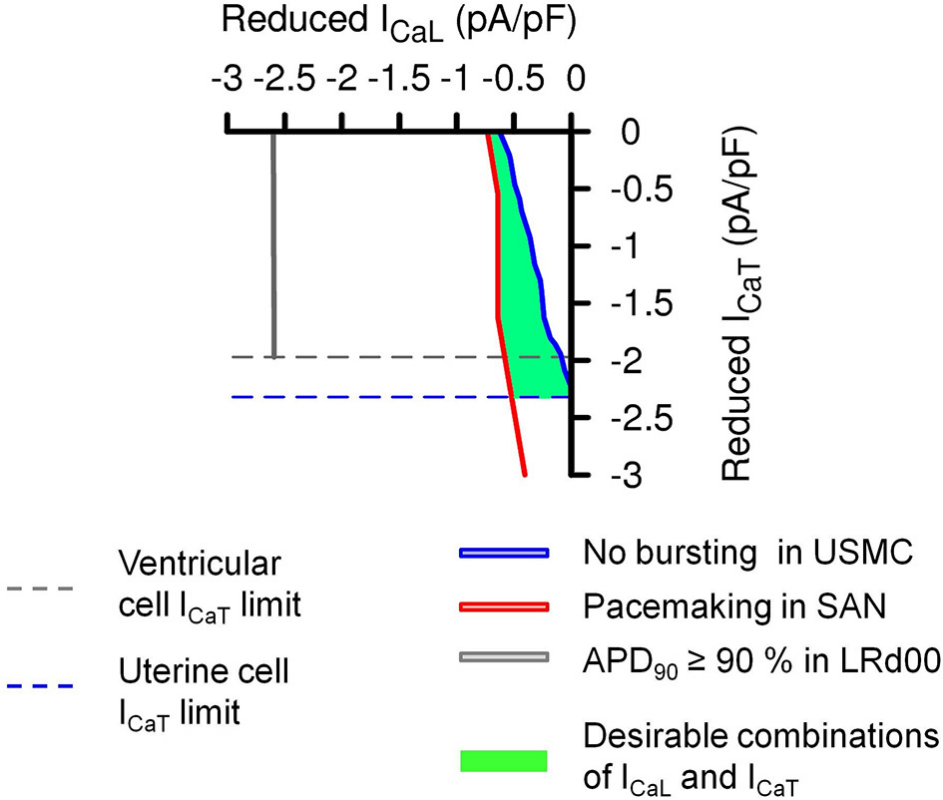
**Effects of the same absolute amounts of I_CaL_ and I_CaT_ reductions on the APs of the three different cell types**. A transformation of the simulation results in Figure 6D using the maximum values from published experimental current densities of I_CaL_ and I_CaT_ (Table 1) in human uterine smooth muscle cells (I_CaL_ = 4.5 pA pF^−1^ and I_CaT_ = 2.32 pA pF^−1^), guinea pig SAN cells (I_CaL_ = 4.6 pA pF^−1^ and I_CaT_ = 5.4 pA pF^−1^), and guinea pig ventricular cells (I_CaL_ = 12.5 pA pF^−1^ and I_CaT_ = 1.97 pA pF^−1^). The dashed lines are the experimental limits of I_CaL_ and I_CaT_ that can be reduced in the different cell types. The solid color lines and the green area represented the same conditions as in Figure 6.

The above experiments have considered the outcomes on AP form when imposing the same proportional reduction of I_CaL_ and I_CaT_ in all three cell types. However, another interesting possibility would be the same absolute quantities of currents being reduced in all three cell types. This consideration arises from examining the range of current densities of I_CaL_ and I_CaT_ reported from the different cell types as noted in Table 1. Cells of the cardiac conduction system tend to have much more I_CaT_ than contractile cardiomyocytes and, when comparing between uterine and cardiac cell types, the total I_CaT_ found in USMCs amounts to around 42% of that reported from guinea pig SAN. We re-performed our simulations to test the effects of reducing the same quantities of currents in each of the three cell type models. This resulted in a transformation of the XY axes of the parameter maps in Figure 6 to current densities and the result is shown in Figure 7. Now, the simulations reveal a larger domain within which one may reduce I_CaL_ and I_CaT_ to produce effective inhibition of USMC model APs with little detriment to cardiac cell model APs. In particular, it is possible to completely block the USMC bursting by large reduction of the USMC I_CaT_ without affecting the SAN pacemaking. Therefore, our first experimental protocol of examining the effects of proportional current reductions likely represents the most stringent case.

**Table 1:** **Summary of I_CaL_ and I_CaT_ current density ranges reported in uterine and cardiac cells**.

**Tissue type**	**Species**	**I_CaL_ (pA pF^−1^)**	**I_CaT_ (pA pF^−1^)**	**References**
Uterus	Rat	1.49–7.17		1–8
	Human	0.65–4.5^*^	0.89–2.32^*^	9–13
SA node	Guinea pig	2.8–4.6	3.0–5.4	14–16
	Mice	4.4–7.6	5.2–8.4	17
	Rabbit	22	2–6	18, 19
	Pig	3.1	n.d.	16
AV node	Mice	3.6–4.6	5	17
Purkinje cells	Canine	2.28–6.1	0.99–4.6	22–24
Atrial cells	Guinea pig	4.66–6.36^*^	2.21–2.55^*^	20, 21
	Mice	6.0–8.0	n.d.	17
Ventricular cells	Guinea pig	4.33–12.5^*^	n.d.–1.97	21, 25–28

*n.d., Not detected*.

* *Some experimental values were reported in pA and they were converted to pA pF^−1^ with published capacitances: late pregnant human myometrium (Cm = 140 pF, Blanks et al., 2007); guinea pig atrial cells (Cm = 44 pF); and ventricular cells (Cm = 115 pF, James et al., 1996)*.

*References: 1, Ohya and Sperelakis, 1989; 2, Inoue and Sperelakis, 1991; 3, Miyoshi et al., 1991; 4, Kusaka and Sperelakis, 1995; 5, Yoshino et al., 1997; 6, Shmigol et al., 1998; 7, Okabe et al., 1999; 8, Jones et al., 2004; 9, Inoue et al., 1990; 10, Young et al., 1991; 11, Young et al., 1993; 12, Knock and Aaronson, 1999; 13, Blanks et al., 2007; 14, Kojima et al., 2012; 15, Kojima et al., 2014; 16, Ono and Iijima, 2005; 17, Mangoni et al., 2006; 18, Hagiwara et al., 1988; 19, Ono and Iijima, 2010; 20, Xu and Lee, 1994; 21, Camara et al., 2001; 22, Hirano et al., 1989; 23, Han et al., 2001; 24, Rosati et al., 2007; 25, Balke et al., 1992; 26, Masumiya et al., 1998; 27, Takeda et al., 2004; 28, Zorn-Pauly et al., 2004*.

## Discussion

Muscle contractions are determined by the form and duration of the preceding APs and the corresponding calcium fluxes. I_CaL_ is involved in the depolarization of APs and is a major source of calcium influx in many excitable cell types including uterine smooth muscle and cardiac muscle cells. As it has been shown in experimental studies (Kodama et al., 1997; Terrar et al., 2007; Lee et al., 2009; Young and Bemis, 2009) and in this study, reducing I_CaL_ affects the amplitude and the duration of the AP and/or contractile force in these cell types. These properties made I_CaL_ an obvious choice as a target for tocolytic treatment. Indeed, nifedipine is used clinically as a second line tocolytic for short periods and within tight dosage recommendations; caution in its use is warranted because of the possibility of side effects including palpitations and hypotension (van Geijn et al., 2005; RCOG, 2011). This alerts one to a necessary consideration of the possible actions on cardiac electrical excitability by tocolytic drugs intended to limit uterine APs.

From our simulations, the USMC model seems to be more susceptible to reduced I_CaL_ than the two cardiac (SAN and ventricular) cell models. However, the influence on the cardiac SAN model of reducing I_CaL_ appears rather steep and so, as one attempts to increase the USMC effects by greater I_CaL_ reductions, there too is a potentially damaging influence on SAN activity and, by extension, heart pacing. This may relate to the side effects on cardiac functions and the tight dosage guidelines recommended for nifedipine use as a tocolytic (RCOG, 2011).

With its potential role in facilitating the onset of USMC APs, I_CaT_ is an alternative tocolytic target. The RMP of USMCs of around −55 mV is close to the voltage level for the window current from I_CaT_ (Blanks et al., 2007). However, experimental studies in cardiac SAN cells illustrate that maneuvres designed to reduce I_CaT_ result in alterations of SAN APs (Hagiwara et al., 1988; Masumiya et al., 1998; Tanaka et al., 2008). In our study, modest reduction of I_CaT_ hyperpolarizes the USMC model RMP and diminishes the bursting spikes with a mild effect on the SAN model cell AP. However, as above for I_CaL_, further reductions in I_CaT_ with the purpose of abrogating USMC model APs resulted in deleterious actions on the SAN cell model APs.

In contrast, our data suggests that reducing both I_CaL_ and I_CaT_ can confer advantages for achieving our objective. This strategy may facilitate delaying/slowing the onset of the USMC APs. In our simulations, this combined approach clearly works more effectively than reducing either of the currents alone. Furthermore, we have identified a range of desirable combinations of I_CaL_ and I_CaT_ inhibition for maximizing the desirable action on USMC model APs without attracting an adverse effect on cardiac SAN or ventricular cell model APs.

If this approach is to work, then there will have to be an agent that blocks I_CaT_ with at least as good selectivity as nifedipine for I_CaL_. Mibefradil has been suggested to be a selective T-type Ca channel blocker but there are several studies indicating that it can affect both L-type and T-type calcium channels (Masumiya et al., 1999; Protas and Robinson, 2000; de Paoli et al., 2002). From such a seeming disadvantage, could one utilize the cross-channel inhibition of a particular drug for the purposes described above? i.e., might the use of one drug eventuate the situation of beneficially inhibiting both I_CaL_ and I_CaT_? Mibefradil, at 1 μM, was shown to block 55% of I_CaT_ in rabbit sinonatrial nodal cells (Protas and Robinson, 2000) but also and 64% of I_CaL_; at 3 μM, it blocked 28% of I_CaT_ and 15% of I_CaL_ in guinea pig ventricular cells (de Paoli et al., 2002); at 10 μM, it inhibited 90% I_CaT_ in guinea pig ventricular cells (Masumiya et al., 1999) and also 40% I_CaL_. If we compare these combinations of proportional inhibitions of I_CaL_ and I_CaT_ against our results in Figure 6, all of these combinations fall *outside* our desired proportional mix of these two currents for a useful tocolytic treatment. Instead, based on our simulation results, if mibefradil was used as tocolytic treatments at these concentrations, it might reduce uterine bursting APs but it would likely affect cardiac pacemaking functions as well. Indeed, mibefradil, at 1 μM, was shown to attenuate the contractile forces of uterine muscle strips from late pregnant rats (IC50 ~1 μM, Lee et al., 2009). However, at the same concentration, mibefradil also reversibly stopped the pacemaking activities from sinoatrial nodal cells (Protas and Robinson, 2000). Thus, it would seem that the dual actions, and limited channel-specific discretion, of mibefradil on I_CaL_ and I_CaT_ would not be advantageous to our purpose in both *in silico* and *in vitro* experimental settings. In addition, it has been reported to have inhibitory actions on a number of non-Ca channels including I_Na(1.5)_, a major Na current in cardiomyocytes (McNulty and Hanck, 2004). This highlights another issue, therefore, in the prosecution of these studies in the longer-term, i.e., that the development of inhibitors with greater selectivity for T-type Ca channels would be a major advance.

In this *in silico* assessment of potential tocolycis, not only did we consider the effectiveness of action on the uterus, but we also assessed the possible side effects to different cells of the heart. This approach is only possible as the computational models of both organs are developed with sufficient biophysical details. The most obvious limitation for this kind of assessment is that it depends on the depth and accuracy of biophysical details in the computational models. We can only include quantitative details from experimental data but often we are forced to make assumptions and educated guesses for the unknowns. Indeed, we have seen in our study that close scrutiny of existing models, however well established, can reveal anomalies that need addressing—in our case the I_CaT_ characteristics in the ventricular cell model—before experimental simulations with particular purposes can be tested. This is the benefit of the iterative process. As computational models continue to be tested, and evolved to incorporate new and relevant biophysical details, the accuracy of the quantitative predictions will improve.

### Future directions

Our work brings to the fore the notion that, instead of searching for a single tocolytic compound with high specificity, there may be merit in considering a cocktail of more than one compound. The present recommendations in the UK are to avoid combinations of tocolytic drugs for fear of increasing the risk of side effects (RCOG, 2011). However, this caution arises from a lack of depth to the background research data and the paucity of currently available tocolytic options. The progression to labor has been described as a modular accumulation of (patho)physiological systems—or MAPS—and it is such modularity that is a major challenge to overcome in seeking to prevent or inhibit labor (Mitchell and Taggart, 2009). The suspicion is that once module A of the labor process is inhibited, there is module B, or module C or more that eventually facilitates a similar function and outcome of labor and delivery. If this is so, then the possibilities offered by combination drug strategies must continue to be explored.

Our assessment only considered effects at the cell level. However, to fully evaluate the actions of any tocolytic compounds, we need to also consider their effects at the tissue and organ levels. Currently there is no validated computational organ model for the uterus that would serve well this purpose although it is recognized by many that efforts toward this are required (Aslanidi et al., 2011a; Sharp et al., 2013). We are under no illusion that there is a need for much more “wet” experimentation—particularly in USMCs—to furnish computational model improvement, and to test with increasing rigor and clarity, important hypotheses of relevance to tocolysis.

## Conclusion

Our approach herein can be regarded as a useful platform to be built upon for assessing the potential of tocolytics that act upon ion channels or electrogenic ion exchangers. Using the *in silico* approach described above enables future research to assess in parallel the potential benefits of attenuating premature uterine contractions vs. the risks of deleterious actions on cardiac electrical excitation.

Encouraging such *in silico* assessments at the early stages of laboratory or clinical studies will foster an integrated and iterative process between mathematical modeling and experimentation such that each informs the other. Related to this, it can alert one to the possibility of otherwise unforeseen actions of drugs and inform the subsequent protocol development for experimentation. Not only will this be beneficial in the examination of new physiological mechanisms and the actions of novel drugs but it will be useful, as indicated in this study, for the investigation of repurposing the application of existing drugs. It is to be hoped that similar approaches may form part of forthcoming scientific and clinical scientific strategies.

### Conflict of interest statement

The authors declare that the research was conducted in the absence of any commercial or financial relationships that could be construed as a potential conflict of interest.
