# Supplementary material for: Computational modeling of inhibition of voltage-gated Ca channels: identification of different effects on uterine and cardiac action potentials
Source: Front Physiol. 2014 Oct 16;5:399. doi: 10.3389/fphys.2014.00399 (PMC4199256; doi:10.3389/fphys.2014.00399)
Supplement: Supplementary file 1 [file Presentation1.PDF]

Appendix

Computational modeling of voltage-gated Ca channels inhibition: identification of different effects on uterine and cardiac action potentials

1. Appendix

Initial conditions used in this study for the USMC model.

USMC Variables	Initial conditions	Descriptions
V	-56.62583251	Membrane voltage
$[Ca^{2+}]_i$	8.54E-05	Intracellular Calcium concentration
m	0.096502568	I_{Na} activation gating variable
h	0.488309397	I_{Na} inactivation gating variable
b	0.43967204	I_{CaT} activation gating variable
g	0.048059534	I_{CaT} inactivation gating variable
d	0.007057741	I_{CaL} activation gating variable
f_1	0.934675559	I_{CaL} fast inactivation gating variable
f_2	0.934675559	I_{CaL} slow inactivation gating variable
q	0.189956989	I_{K1} activation gating variable
r_1	0.26663309	I_{K1} fast inactivation gating variable
r_2	0.26663309	I_{K1} slow inactivation gating variable
p	0.103044656	I_{K2} activation gating variable
k_1	0.998004889	I_{K2} fast inactivation gating variable
k_2	0.998004889	I_{K2} slow inactivation gating variable
x_{α}	0.000425792	$I_{BK(Ca)\alpha}$ activation gating variable
$x_{\alpha\beta1}$	0.001213076	$I_{BK(Ca)\alpha\beta1}$ activation gating variable
s	0.021684704	I_{Ka} activation gating variable
x	0.122639549	I_{Ka} inactivation gating variable
y	0.003561435	I_h activation gating variable
c	0.000225331	$I_{Cl(Ca)}$ activation gating variable

Initial conditions used in this study for the SAN model.

SAN		

Variables	Initial conditions	descriptions
V	- 39.013558536	Membrane voltage
m	0.092361701692	I_{Na} activation gating variable
h_1	0.015905380261	I_{Na} fast inactivation gating variable
h_2	0.01445216109	I_{Na} slow inactivation gating variable
d_L	0.04804900895	I_{CaL} activation gating variable
f_L	0.48779845203	I_{CaL} inactivation gating variable
d_T	0.42074047435	I_{CaT} activation gating variable
f_T	0.038968420558	I_{CaT} inactivation gating variable
y	0.03889291759	I_f activation gating variable
r	0.064402950262	I_{to} activation gating variable
q	0.29760539675	I_{to} inactivation gating variable
$p_{a,f}$	0.13034201158	I_{Kr} fast activation gating variable
$p_{a,s}$	0.46960956028	I_{Kr} slow activation gating variable
p_i	0.87993375273	I_{Kr} inactivation gating variable
x_s	0.082293827208	I_{Ks} activation gating variable

Initial conditions used in this study for the LRd00 model.

LRd00 Variables	Initial conditions	descriptions
V	-87.46754137	Membrane voltage
m	0.00102309	I_{Na} activation gating variable
h	0.991296132	I_{Na} fast inactivation gating variable
j	0.99426209	I_{Na} slow inactivation gating variable
d	4.06E-06	I_{CaL} activation gating variable
f	0.998871307	I_{CaL} inactivation gating variable
b	0.004714259	I_{CaT} activation gating variable
g	0.796900605	I_{CaT} inactivation gating variable
xr	0.00019515	I_{Kr} activation gating variable
$xs1$	0.028741432	I_{Ks} activation gating variable
$xs2$	0.097512826	I_{Ks} inactivation gating variable
$[Na^+]_i$	16.39085888	Intracellular Sodium concentration
$[Na^+]_o$	139.8976397	Extracellular Sodium concentration
$[K^+]_i$	133.1224874	Intracellular Potassium concentration
$[K^+]_o$	4.500305727	Extracellular Potassium concentration
$[Ca^{2+}]_i$	0.000392292	Intracellular Calcium concentration
$[Ca^{2+}]_{jsr}$	5.5002723	Calcium concentration in JSR compartment

$[Ca^{2+}]_{nsr}$	5.449829462	Calcium concentration in NSR compartment
$[Ca^{2+}]_{o}$	1.855215919	Extracellular Calcium concentration

Formulation of a new T-type calcium current for ventricular cell:

$$I_{\mathrm{CaT}} = \bar{g}_{\mathrm{CaT}}\ b^2\ g\ (V - E_{\mathrm{CaT}}) \tag{1}$$

$$E_{\mathrm{CaT}} = \frac{R\ T}{z\ F}\log\left(\frac{[\mathrm{Ca}^{2+}]_{\mathrm{o}}}{[\mathrm{Ca}^{2+}]_{\mathrm{i}}}\right) \tag{2}$$

$$b\infty(V) = \frac{1}{1+\exp\left(-\frac{\mathrm{V}+50}{5}\right)} \tag{3}$$

$$g\infty(V) = \frac{1}{1+\exp\left(-\frac{\mathrm{V}+61}{5}\right)} \tag{4}$$

$$\tau_b(V) = \frac{5}{1.068\exp\left(\frac{\mathrm{V}+16.3}{30}\right)+1.068\exp\left(-\frac{\mathrm{V}+16.3}{30}\right)} \tag{5}$$

$$\tau_g(V) = \frac{5}{0.015\exp\left(\frac{\mathrm{V}+71.7}{83.3}\right)+0.015\exp\left(-\frac{\mathrm{V}+71.7}{15.4}\right)} \tag{6}$$

$$\frac{db}{dt} = \frac{b\infty(V)-b}{\tau_g(V)} \tag{7}$$

$$\frac{dg}{dt} = \frac{g\infty(V)-g}{\tau_g(V)} \tag{8}$$

where $\bar{g}_{\mathrm{CaT}} = 0.056\ \mathrm{nS\ pF}^{-1}$. R is the universal gas constant, T is temperature and F is the Farady constant, z is the calcium ion valency. The modified I_{CaT} consists of one activation (b) and one inactivation (g) variables (Eq 1). E_{CaT} is the reversal potential for calcium (Eq 2). Equations 3 and 4 are the activation and inactivation steady-state functions. Equations 5 and 6 are the activation and inactivation time constants functions. The voltage and time dependent characteristic of the activation and the inactivation were described by the Hodgkin-Huxley type differential equations (Eq 7-8).
